# The Impact of Placement Change on Sleep in Child Welfare

**DOI:** 10.3390/children13050631

**Published:** 2026-05-01

**Authors:** Haritomane Brillakis, Xiaoran Tong, John S. Lyons

**Affiliations:** 1 Center for Public Health Services and Systems Research, College of Public Health, University of Kentucky, Lexington, KY 40506, USA; 2 Department of Psychiatry & Behavioral Neurosciences, University of Chicago, Chicago, IL 60637, USA; xiaoran.tong.cn@gmail.com

**Keywords:** child welfare, placement instability, foster care, youth, sleep disturbance

## Abstract

**Highlights:**

**What are the main findings?**

- Sleep disturbance increased over time during follow-up of youth in foster care, rising from 9.65% with moderate or severe sleep disturbance at baseline, to 13.80% by the end of the follow-up period.
- Greater placement instability was associated with progressively higher subsequent sleep disturbance, even after accounting for sleep at placement entry, demographic characteristics, and baseline psychosocial and clinical needs.

**What is the implication of the main finding?**

- Sleep disturbance may serve as a practical and clinically meaningful indicator of stress and adjustment difficulties during foster care transitions.
- Early sleep screening and targeted support during placement changes may help identify youth at elevated risk and improve monitoring within child welfare systems.

**Abstract:**

**Background/Objectives:** Sleep disturbance is common among youth in the child welfare system, yet the role of placement instability and placement setting in shaping sleep outcomes remains understudied. This study examined the association between placement instability, time spent in different care settings, and sleep disturbance among children in foster care. **Methods:** We conducted a retrospective cohort study using longitudinal administrative child welfare data from a Midwestern U.S. state, including 20,888 youth aged 5–18 years who entered foster care between 2010 and 2020. Sleep disturbance was assessed using the Child and Adolescent Needs and Strengths (CANS) sleep item. Baseline was defined as the first CANS assessment within one month of entry into care, and follow-up as the assessment closest to discharge or the end of a three-year observation window, whichever occurred first. We estimated association using a time-lagged linear mixed-effects model predicting sleep disturbance after each placement episode, including placement instability: 1 (reference), 2, 3, or ≥4 placement(s), time since placement, time spent in care settings (kinship, foster home, treatment foster home, congregate care, institutional care), and baseline trait factor scores derived from non-sleep CANS items, while controlling for sleep at the time of placement and demographics. **Results:** At baseline, 2016 children had actionable sleep disturbance (CANS sleep = 2 or 3; 1701 moderate and 315 severe). By the end of follow-up, this increased to 2884 children (2372 moderate and 512 severe). In linear mixed-effects models, placement instability demonstrated a dose–response association with higher subsequent sleep disturbance relative to one placement (2 placements: *β* = 0.025; 3 placements: *β* = 0.045; ≥4 placements: *β* = 0.067; all *p* ≤ 0.02). Time spent in kinship care was associated with lower sleep disturbance (*β* = −0.049; *p* < 0.001), whereas time spent in treatment foster homes was associated with higher sleep disturbance (*β* = 0.035; *p* < 0.001). Trauma in the family, medical/developmental needs, and internalizing/sexual issues were positively associated with sleep disturbance. Time and instability interactions showed modest attenuations of instability-associated sleep disturbance over time for higher placement counts. **Conclusions:** Placement instability is associated with progressively worse sleep disturbance over time among youth in foster care, even after controlling for sleep status at placement and baseline functioning. Sleep disturbance may represent an actionable indicator for the child welfare system, highlighting opportunities for targeted screening and support during placement transitions.

## 1. Introduction

Sleep is a foundational biological and developmental process, and disturbances during childhood can have consequences that extend into adulthood, affecting emotional regulation, behavior, and long-term health. Insufficient or irregular sleep in early childhood has been associated with poorer adjustment and daytime behavioral problems, underscoring the intertwined nature of sleep health and psychosocial functioning [1,2]. Children and adolescents involved in the child welfare system are particularly vulnerable to sleep disturbances, with studies consistently reporting a higher prevalence of delayed sleep onset, frequent awakenings, nightmares, and irregular sleep schedules among foster care youth compared with their peers [3,4,5,6]. In a nationally representative study of U.S. children in the child welfare system, approximately 62% of youth exhibited a clinically meaningful pattern of sleep disturbance, with 47% endorsing trouble maintaining sleep, and 16% reporting oversleeping and daytime fatigue [7]. Prior studies have similarly documented prolonged sleep latency and disrupted sleep patterns, further supporting vulnerability of this population to sleep-related difficulties [8,9].

These elevated rates are unsurprising given the high burden of trauma, maltreatment, and instability experienced by children prior to, and during, involvement with child welfare systems. Sleep disturbance is both a symptom and a contributing factor to a range of psychiatric conditions, including anxiety, depression, and post-traumatic stress, while poor sleep itself exacerbates emotional dysregulation, learning problems, and impaired attention [1,10,11,12,13]. In this context, the relationship between sleep and mental health is likely bidirectional: traumatic stress and emotional dysregulation may contribute to sleep disturbance, while disrupted sleep may intensify behavioral and emotional difficulties that complicate placement adjustment and service needs.

Children in the child welfare system face many challenges in adjusting to new foster homes and living arrangements. Adapting to unfamiliar environments presents unique obstacles, compounding the challenges they bring to their new homes [14,15]. Unsuccessful integration into new foster homes has been associated with adverse mental health and sleep outcomes among children in foster care [3,5,13]. These factors may also negatively impact children’s sleep quality. Although the literature regarding sleep quality among youth in foster care is gradually expanding, at the time of writing, limited research has examined the specific influence of placement change on sleep health in this population [8,16].

From a child welfare perspective, sleep disturbance is also a highly actionable concern [9,17]. Sleep problems are routinely assessed within child welfare systems using standardized tools such as the Child and Adolescent Needs and Strengths (CANS) assessment and may inform placement decisions, service referrals, and trauma-informed care planning [17,18,19]. Unlike many long-term outcomes, sleep disturbance can signal acute stress during transitions and may be responsive to targeted support, caregiver coaching, and stabilization efforts [9,16]. Understanding whether placement instability and placement type are associated with sleep disturbance may therefore help agencies identify opportunities for earlier monitoring and intervention during periods of heightened vulnerability.

To address this research gap, we studied a large cohort of children and youth residing in the child welfare system of a large Midwestern state. The primary objective was to examine whether placement instability is associated with the likelihood of sleep disturbance over time. We hypothesized that greater placement instability would be associated with higher sleep disturbance at follow-up and over time. We also examined whether time spent in specific placement settings (kinship care, foster home, treatment foster home, congregate care, institutional care) was independently associated with sleep outcomes after adjusting for sleep at placement, demographic characteristics, and baseline functional needs.

### Conceptual Framework

This study is guided by developmental, trauma-informed, and attachment-based frameworks that link placement instability and placement restrictiveness to sleep disturbance among children involved in the child welfare system. Repeated placement transitions may represent cumulative stress exposure, consistent with models of toxic stress that describe how persistent disruptions in caregiving environments can dysregulate neurobiological systems governing arousal, stress responsivity, and sleep–wake regulation [20,21,22,23].

Attachment theory further posits that stable caregiver relationships provide a critical foundation for nighttime regulation and perceived safety, whereas placement disruptions may undermine attachment security and contribute to heightened nighttime vigilance, anxiety, and sleep fragmentation [24,25,26,27]. Placement settings may also capture differences in contextual demands and underlying behavioral or emotional needs, as youth in more restrictive or service-intensive settings often have higher levels of trauma in exposure and functional impairment [15,28,29].

These processes may vary developmentally. Younger children may be particularly sensitive to disruptions because they rely more heavily on caregiver-mediated regulation of stress and sleep, whereas older youth may exhibit different coping responses shaped by developmental maturation and cumulative adversity [1,27,30].

## 2. Materials and Methods

### 2.1. Participants and Setting

We conducted a retrospective cohort study using longitudinal administrative child welfare data from a large Midwestern U.S. state. The initial dataset comprised 29,915 children with 138,345 Child and Adolescent Needs and Strengths (CANS) assessments conducted between 1 February 2010 and 31 December 2020. Children were followed retrospectively from their initial entry into out-of-home care to discharge (e.g., reunification, adoption, exit) or the right censoring point.

Baseline was defined as the first CANS assessment administered within one month of entry into foster care, and the endpoint is the last documented CANS assessment—considering right censoring and premature discharge. Permission to access the de-identified study population data was granted by the child welfare authority of the large Midwestern state.

After data selection and quality control procedures (see Appendix A and Appendix B and Supplement “Data Example”), the working sample included 20,888 children aged 5–18 years who entered out-of-home care during the study period. The analytic sample included 90,827 CANS assessments of 20,888 individuals aligned with 43,094 placement episodes lasting at least 30 days, spanning kinship care, foster homes, treatment foster homes, congregate care, institutional care, and exit-related placement categories.

### 2.2. Measures

The measurements are longitudinal and organized hierarchically: each CANS assessment represents the most granular observation, assessments are grouped within placement periods, and placement periods are grouped within individuals (See Supplement “Data Example”).

#### 2.2.1. CANS Tool

The Child and Adolescent Needs and Strengths (CANS) is a structured, consensus-based assessment tool designed to communicate the wellness of a child and their family. In child welfare, CANS is typically administered by certified workers to monitor children on a routine basis, or upon significant changes in the status of the child, such as removal and initial placement into out-of-home care, change of placement, and the end of care [17,19]. CANS has become widely used across all 50 states in the US and multiple countries [18,19] The tool’s primary objectives are to support decision-making processes, promote quality improvement, and monitor outcomes within the child-serving system under the Total Collaborative Outcomes Management (TCOM) conceptual framework [19]. The version of CANS used in this study comprises 150 items spanning multiple functional and clinical domains related to the child and the caregivers’ needs and strengths, which is mandated to be administered at a 6-month interval to youths in out-of-home care [31] (see Supplement “Dictionary of CANS”), and the caseworkers using the tool are mandated to be re-trained and re-certified on an annual basis [31]. In practice, the caseworker reaches consensus with the primary caregivers on the ratings for each item; optionally, when the youth is also present during the CANS assessment, the consensus on each item extends to the youth. The items are rated at 4 action levels of “0”, “1”, “2”, and “3”. For needs-based items, a rating of “0” indicates no evidence of a need, “1” indicates a history or concern that a need may exist, “2” indicates a moderate need, and “3” indicates an intensive need that is either dangerous or disabling. Prior research has demonstrated that CANS items are reliable and valid as individual indicators (*) [17]. Anderson et al. reported a strong interrater reliability of 0.85 on the Life Functioning domain among researchers, and an intraclass correlation of 0.81 between caseworkers and researchers. They also reported that most rating differences occurred between a rating of “0” and “1” or between a “2” and “3”, which generally does not affect care planning since agencies typically act upon items rated “2” or “3”. Therefore, it is common for studies to dichotomize CANS items rated “1” or “2” as “non-actionable “ (rating of 0 or 1 = 0), and = “actionable” (ratings of “2” or “3” = 1) [17].

#### 2.2.2. Sleep Disturbance

Sleep disturbance was measured by the “sleep” item from the CANS Life Functioning domain. The item captures sleep-related problems including difficulty falling asleep, excessive sleeping, bed-wetting, and nightmares, regardless of underlying causes: accordingly, sleep disturbance is interpreted as a marker of a broader nighttime dysregulation rather than an isolated sleep disorder. The 4 action levels of “Sleep” are as follows:

A rating of 0: There is no evidence of problems with the sleep process. The child gets a full night’s sleep each night.

A rating of 1: The child has some problems sleeping and generally gets a full night’s sleep, but problems may arise once a week. This may include occasionally awakening or bed-wetting or having nightmares.

A rating of 2: The child is having problems with sleep. Sleep is often disrupted, and the child seldom obtains a full night of sleep.

A rating of 3: The child is sleep-deprived. Sleeping is almost always difficult, and the child is not able to get a full night’s sleep which prevents them from going to school or engaging with peers.

Alternatively, a rating of “2” or “3” on the item can be seen as an “actionable” sleep disturbance, and a rating of “0” or “1” as “non-actionable” sleep disturbance, indicating no, or minimal, sleep difficulty. In this study, the “non-actionable” vs. “actionable” sleep disturbance categorization serves as the sensitivity analysis. Sleep is treated as either the primary outcome or one of the core predictors, depending on the timing of the assessment. We exclude the first assessment conducted on or near the day of placement and use only the “Sleep” item from the CANS collected a while after the placement date. We currently define this as after 30 days, based on our observation of a marked increase in the number of follow-up assessments occurring beyond 30 days after the initial assessment conducted at or near the beginning of a placement.

#### 2.2.3. Placement

Placement instability as the primary predictor was operationalized as the cumulative placement count (CPC) since entering into the system, until the conclusion of out-of-home care or right censoring. Placement instability was categorized into 4 levels: one placement as the reference category (no instability), two placements as moderate instability, and three placements or four or more placements indicating high instability.

Placement type as the secondary predictor was captured by 6 categories: kinship care, foster home care, treatment foster home (TFH) care, congregate care (i.e., group homes and shelters), institutional care (i.e., detention), and a special category of exiting placement (i.e., trial reunification and supervised independent living). Since individuals often move between settings over time, we therefore express the dynamics as 6 vectors of cumulative placement type (CPT), where each vector records the years spent in one category so far at the date of a CANS assessment.

#### 2.2.4. Covariates

The covariates included age at entry (5–9, 10–15, 16–18 years), gender (male and female), race (N/A, White, Black, Native Alaskan/American Indian, Native Hawaiian/Pacific Islander, and Asian), ethnicity (N/A, Hispanic, and non-Hispanic). The “N/A” category in race and ethnicity represents “Refused to Report/Unknown” and will be treated as the reference level to preserve analytical sample size. Implicitly, the cumulative years spent in out-of-home care is also a covariate, which is the sum of the 6 cumulative placement type vectors.

#### 2.2.5. Baseline Traits

To adjust for clinical and functional traits, prior to entering out-of-home care that could affect both sleep and placement, we conducted factor analysis to summarize interpretable factor scores from the CANS items, other than the “Life Function/Sleep” item. Out of the 149 items (i.e., 150 items minus “Sleep”) from 15 domains, we retained 92 from 9 mandatorily assessed core domains (missing < 0.2%) for the factor analysis (See Section 2.3.2. in Section 2 and Section 3.2.1 in Section 3).

### 2.3. Statistical Analysis

We conducted a longitudinal analysis using a linear mixed model (LMM) with R v4.1.2 Statistical Software [32] to examine the hypothesized determinants of sleep disturbance after a placement date, with the primary focus on the impact of increased placement instability, followed by changing placement types, prior individual traits, and demographics.

#### 2.3.1. Time-Lagged Longitudinal Analysis by Linear Mixed Model

To examine the association between placement experiences and subsequent sleep disturbance, we estimated a time-lagged linear mixed-effects model. This approach allows for repeated measurements of sleep within individuals across placement periods while accounting for both within- and between-person variation.

Formally, the fixed effect portion of the model is

$$y_{ijk}=x_{i}+BLT_{i}+y_{ij0}+CPC_{ij}+\;CPT_{ijk}+\tau_{ijk}+CPC_{ij}\tau_{ijk}$$

The left-hand side “*y*” is the sleep status at the *k*th assessment (at least 30 days after placement date) for the *j*th placement of the *i*th individual.

On the right-hand side, the term “*x*” denotes nominal variables of age, gender, race and ethnicity categories for the *i*th individual. “BLT (baseline traits)” contains 7 factor scores calculated from rest of the baseline CANS core domain items other than “Sleep” for the *i*th individual; “*y*” again denotes sleep status, but its special subscript *k* = 0 indicates the timing of assessment being at or near the beginning of the *j*th placement; the term “CPC (cumulative placement count)” expands to 3 nominal variables flagging the *j*th placement as being the 2nd, 3rd, or 4th+ placement for the *i*th individual (as opposed to a stable placement); “CPT (cumulative placement type)” expands into 6 vectors of years spent in each of the 6 placement settings right before the *k*th assessment past the *j*th placement for the *i*th individual; “*τ*” is the years of the *k*th assessment lagged behind the *j*th placement of the *i*th individual.

The fixed coefficient (i.e., *β*) on right-hand side term “*y*” captures the autoregressive effect of sleep at the beginning of a placement on sleep in later periods of the same placement, as well as the reverse causal effect of sleep on the decision of said placement. Consequently, the “leftover” effect of placement instability on sleep identified by the coefficients of time can be considered causal; that is, the expected change in sleep disturbance (at least 30 days after placement date) after 2, 3, or 4+ placements in comparison to staying in one placement. Similarly, the coefficients of “type” represent the causal effect of cumulatively staying in one of the 6 placement settings. Lastly, the coefficients of interaction terms “Time × *τ*” capture either an amplifying or diminishing effect of placement instability on sleep overtime.

The random effect portion of the model is

$$z_{ijk}=a_{i}+a_{ij}+b_{ij}\tau_{ijk}$$

The two random intercepts “*a*” capture varying baseline sleep status specific to every individual entering out-of-home care and to the beginning of every placement nested within an individual; the random slope “*b*” captures the varying trajectory of sleep disturbance over time elapsed (in years) specific to each placement period within each individual. Consider the difficulty of convergence for a linear mixed model. We reserved two simpler random components: one lacks the random slope at the placement level; the other lacks both random intercept and random slope at the placement level. On the other hand, we also reserved a more complex random component with one additional random slope on elapsed time since each placement, but varying at the individual level.

Ideally, the “Sleep” outcome rated in discrete categories of “0”, “1”, “2”, and “3”, incrementally, ought to be treated as a 4-level ordinal variable or a 3-trial binomial, fitted by a cumulative link mixed model (CLMM, R-package “ordinal”) or a generalized linear mixed model (GLMM R package “lme4”), respectively. Alternatively, we also dichotomize the outcome as “non-actionable” vs. “actionable” and fit it again with a generalized linear mixed model. Both CLMM and GLMM are more appropriate than LMM since the outcome is not a normal continuous variable, but both are harder to converge.

The final analytical data for the above procedures contained M = 46,826 assessments on N = 14,184 individuals, which was smaller than the full dataset (M = 90,827, N = 20,888), because (1) the initial assessment at/near the beginning of each placement was dropped, except the sleep status, which was stripped and broadcasted to the rest of the assessments to capture the autoregressive effect within the same placement period; (2) we had to drop entire placement periods without an initial assessment at/near (≤ 30 days) the beginning, because they cannot model the autoregressive effect. However, the full data was still required to calculate the 6 cumulative placement types, which also allow us to summarize the primary placement type for every individual.

#### 2.3.2. Factor Analysis for Baseline Traits

We conducted an exploratory factor analysis (EFA) to summarize major dimensions of individual traits based on CANS profiles, except the “Sleep” item. We started with 92 items from core domains that have near-zero missing rates. To better utilize the data, we recycled the initial assessments of each person unused by the longitudinal analysis, which gave 20,888 records.

We dropped 3 items with non-zero response rates lower than 5% (1.5% for “Traumatic Experience: Natural or Manmade Disaster”, 1.8% for “Life Functioning: Expectant Parent or Parenting”, and 0.6% for “Caregiver Needs: Developmental”). We then iteratively performed the Kaiser, Meyer, and Olkin (KMO) test and removed items with the lowest Measure of Sampling Adequacy (MSA) value until the minimum MSA among remaining items was greater than 0.70 (i.e., “middling” adequate); 2 items were removed (Traumatic Experience: Medical Trauma, and Behavioral & Emotional Needs: Substance Use). The polychoric correlation among the remaining 87 items was positive-definite, with a minimum eigenvalue of 0.055.

**Figure 1:**
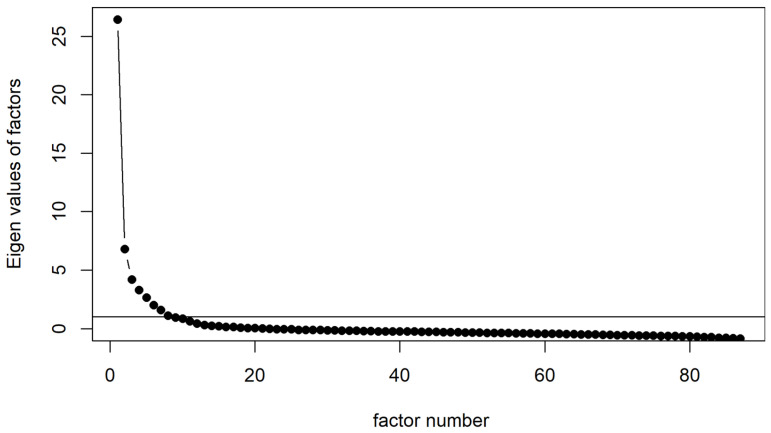
Scree plot of 87 CANS items for factor analysis. Description: The Scree plot created by the R package “psych” is based on 92 items from CANS core domains—except for the “Sleep” item. The plot suggested 8 factors (i.e., the number of eigenvalues of the 92-item correlation that were greater than 1.0). The dots represent 92 eigenvalues sorted from the largest to the smallest along the x-axis; the horizontal line highlight the threshold of eigenvalue = 1 on the y-axis.

The Scree plot (Figure 1) suggested 8 factors. We used the R package “psych” to perform factor analysis on the 87 items with oblique rotation and up to 8 factors. Given an output loading matrix, we considered an item primarily loaded on a factor if (1) the loading on said factor was the largest of the said item and greater than 0.35, and (2) the second largest loading of the same item was at least 0.10 smaller than the largest loading (i.e., a low ambiguity). After flagging the primary loadings, we considered a factor being well-defined if (1) the said factor had 4 or more items primarily loaded on it, and (2) the Cronbach Alpha of the primarily loaded items was higher than 0.80 (i.e., good internal consistency). If any of the factors in a solution were not well-defined, we reduced the number of factors by 1 and re-ran the analysis until all factors are satisfactory. Lastly, we interpreted the latent dimensions measured by each factor according to the primary loaded CANS items.

As we were given an interpretable factor solution, we calculated and treated the factor scores as entry-point latent traits per individual; we then inserted the “traits” as predictors into the main analysis. In Section 3, we report the factor analysis before the main analysis.

## 3. Results

### 3.1. Descriptive Characteristics by Placement Instability and Placement Setting

Table 1 (and Supplement “Population Characteristics”) presents demographic and placement-duration characteristics for 20,888 children stratified by total placement count, ranging from one stable placement (*n* = 10,371) to high placement instability (7–22 placements; n = 542).

Higher placement counts were associated with older entry age and there were substantial racial disparities (Table 1). Entry age differed significantly across placement-count groups (*p* < 0.001), with median entry age increasing from 11 years among youth with 1–3 placements to 12 years among those with ≥4 placements.

Age distribution also varied by total placement count (*p* < 0.001). Among children with a stable placement, 44.9% entered care at ages 5–10, 31.5% at ages 11–15, and 23.6% at ages 16–18. In contrast, among children with 7–22 placements, the distribution shifted toward early adolescence, with 38.2% entering at ages 5–10, 57.9% at ages 11–15, and 3.9% at ages 16–18. The racial composition varied substantially across placement-count groups (Fisher exact *p* < 0.001). In the stable placement group, 61.4% of children were White and 28.6% were Black, whereas among children with 7–22 placements, 36.9% were White and 57.0% were Black. American Indian/Alaska Native youth comprised 6.3% of the overall cohort, with relatively consistent proportions across placement-count strata (range: 5.5–7.2%).

We observed shorter average placement durations as placement instability increased (*p* < 0.001). The average length of individual placements decreased with increasing placement count, from a mean of 0.956 years (SD 0.938) among youth with stable placements to 0.999 years (SD 0.745) among those with 3 placements, and 0.767 years (SD 0.463) among those with 7–22 placements, respectively. Conversely, the total time in care increased sharply with placement instability: mean total length of placement time rose from 0.956 years (SD 0.938) in the stable group to 5.41 years (SD 1.99) among youth with 7–22 placements (*p* < 0.001).

Primary placement type also displayed distinct demographic profiles. Youth in congregate and institutional settings entered care at substantially older ages and were disproportionately male, compared with youth in kinship and foster homes who entered at younger ages (Table 2, and Supplement “Population Characteristics”). Placement duration differed by setting, with institutional and congregate care showing shorter average placement lengths, and treatment foster care showing longer total time in care (Table 2).

At baseline, the majority of youths had no evidence of sleep problems (n = 15,178), while n = 3694 had mild sleep problems (sleep = 1), n = 1701 had moderate sleep problems (sleep = 2), and n = 315 had severe sleep problems (sleep = 3) (Table 3 and Supplement “Population Characteristics”).

Age distributions differed significantly by baseline sleep status (χ^2^ = 43.7, *p* < 0.001). The proportion of youth aged 16–18 was noticeably higher among those rated “2” on sleep (25.3%) than that of the overall population (19.9%).

The (within-person) average length of placement differed significantly by baseline sleep status (χ^2^(12) = 64.8, *p* < 0.001); the proportion of youth who experienced placements of 3 years or lengthier (11.1% + 6.3%) was higher among those who started with severe sleep disturbance at the baseline than in the overall population (6.4% + 3.0%). The same pattern was observed for the total length of placement.

By the end of follow-up, n = 13,450 youth had no evidence of sleep disturbance, n = 4554 had mild sleep disturbance (sleep = 1), n = 2372 had moderate disturbance (sleep = 2), and n = 512 had severe disturbance (sleep = 3) (Table 4 and Supplement “Population Characteristics”). We therefore observed an overall increase in sleep disturbance at end of follow-up, or 9.6% rated “2” or “3” on sleep at baseline, compared to 13.8% by the end of follow-up.

Age is again distributed differently by sleep status at the end of follow-up (χ^2^ = 59.6, *p* < 0.001), but in the opposite direction to baseline. The proportion of youth aged 16–18 was noticeably fewer among those with actionable and severe sleep disturbance (18.4% sleep = 2, and 14.1% sleep = 3) than that of the overall population (19.9%).

Unlike those at baseline, race showed disproportionality across levels of sleep disturbance by the end of follow-up (Fisher exact *p* < 0.001). Among individuals with sleep disturbance rated as actionable (i.e., sleep = 2 or 3), the proportions of White youth (55% and 47.9%) were lower than the overall proportion (58.1%), but for Black youth, the proportions (36.4% and 45.7%) were higher than the overall proportion (32.6%).

Regarding the relationship between the length of placements and sleep disturbance at the end of follow-up, a trend similar to that of the baseline was observed; youths with severe sleep disturbance by the end of follow-up tend to have experienced lengthier placements (for average length F = 127.9, *p* < 0.001; for total length, F = 315, *p* < 0.001).

### 3.2. Analytical Results

#### 3.2.1. Exploratory Factor Analysis for Baseline Traits

Figure 1 shows the Scree plot of the 87 CANS items reviewed. Based on the Scree plot, an eight-factor solution was initially considered. However, the EFA retained seven factors, because the eight-factor model produced one inadequately defined factor with fewer than four primary loadings. In total, the seven factors explained 55% of the variance in the original 87 items, and the highest correlation among the factors was 0.53. Of the seven factors, the minimum Cronbach alpha achieved by the primarily loaded items was 0.81, indicating “good” internal consistency (Supplementary Materials: Result of Factor Analysis; Table 5).

According to the primary loadings, the seven factors can be interpreted as Externalizing Behavior, Caregiver, Trauma in Family, Child Strength, Medical/Developmental, Cultural, and Internalizing Behavior. The EFA reported an acceptable RMSEA of 0.078 but an inadequate CFI of 0.71, indicating a lack of fit (Supplementary Materials: Result of Factor Analysis). However, since the goal of the EFA is not to design unidimensional measurements for external use, but to summarize the latent dimensions of individual characteristics for the study population, we did not drop items of small loading to pursue high CFI, but simply calculated seven factor scores as baseline traits (BLT) for every individual, which in turn acted as confounding predictors in the main analysis.

#### 3.2.2. Time-Lagged Effect of Placement on Sleep

The proposed linear mixed model converged within seconds. When treating the outcome as ordinal and binomial, the corresponding cumulative and generalized mixed model could not converge. Therefore, we returned to treating the sleep disturbance outcome as a numerical variable. When comparing with the two simplified random components (i.e., lacking random slope or random intercept at placement level) with the proposed model, the likelihood ratio test gave Chi-square values of 13,477 (Df = 3) and 6908 (Df = 2), respectively, showing significantly better fit of the default model; when comparing with the more sophisticated random component (i.e., adding random slopes at the individual level), the Chi-square value was 5.5 (Df = 3). We therefore focus on interpreting the default model.

Table 6 and Supplement “LMM Results” presents results from the time-lagged mixed-effects model predicting sleep disturbance measured after the beginning of each placement. Sleep disturbance at the time of placement (Sleep_0) showed a strong autoregressive effect on subsequent sleep disturbance (*β* = 0.816, 95% CI: 0.807–0.825; *p* < 0.001), indicating substantial persistence of sleep problems or the lack thereof across a placement period. Noticeably, the time since placement (*τ*) was positively associated with sleep disturbance (*β* = 0.052, 95% CI: 0.034–0.069; *p* < 0.001).

**Figure 2:**
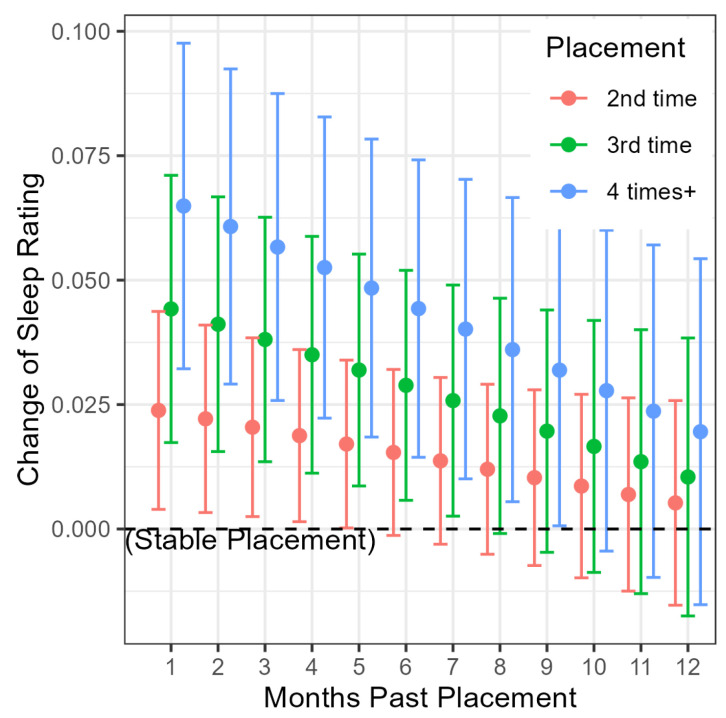
Projected effect of placement instability on sleep. Description: The projected effects of increasingly unstable placement (red, green, and blue) on ratings of sleep disturbance (*y*-axis) up to 12 months (*x*-axis) past the day of placement—versus the reference scenario of single, stable placement (horizontal dash line). To allow the projection and comparison, a reference demographic and a placement setting are implicitly chosen, which were a White non-Hispanic male, aged 5–12 upon entering care, placed into a foster home. The time after the placement was chosen to be between 1 month and a whole year (*x*-axis), as the analysis enforced that an observation must have at least 30 days lag after the day of placement (to allow causal inference), and the third quartile of lag after a placement (but before the next placement or exit) is close to 12 months. The projection is based on key regression coefficients reported by the time-lagged longitudinal model (see Table 6). In particular, (1) the effects of the 3 levels of placement instability (2, 3, and 4+ placements) relative to stable placement are taken from the 3 coefficients of cumulative placement count (CPC), which is shown as an increasingly higher intercept of the 3 colored lines (red < green < blue) and (2) the effect of unstable placement gradually weakens over time as the child adapts to their environment and the attenuation is captured by the 3 regression coefficients on interaction terms between time lag (behind the day of placement) and the level of placement instability, which is shown as negative slopes of the 3 colored lines.

Placement instability as cumulative placement count (CPC) demonstrated a graded effect where higher levels of instability (relative to a single placement) showed higher ratings of sleep quality (2 placements *β* = 0.025, 95% CI: 0.004–0.046; *p* = 0.020, 3 placements *β* = 0.045, 95% CI: 0.017–0.074; *p* = 0.002, and 4+ placements *β* = 0.067, 95% CI: 0.033–0.101; *p* < 0.001). The effects are shown in Figure 2 as projected trends of sleep disturbance past increasingly unstable placement.

Different placement settings—as shown by the 6 cumulative placement types (CPTs), demonstrated different impacts on sleep disturbance. Staying in kinship care is associated with lower sleep disturbance at a later assessment (per year of stay: *β* = −0.049, 95% CI: −0.066 to −0.032; *p* < 0.001), while living in treatment foster homes showed the opposite (*β* = 0.035, 95% CI: 0.023–0.047; *p* < 0.001); spending a year in congregate care was also associated with lower sleep disturbance (*β* = −0.045, 95% CI: −0.060 to −0.031; *p* < 0.001).

Among the seven traits at baseline, “Trauma in Family” was associated with worsened sleep during out-of-home care (per-unit increase in factor score: *β* = 0.023, 95% CI: 0.016–0.030; *p* < 0.001), as were “Medical/Developmental” (*β* = 0.028, 95% CI: 0.019–0.037; *p* < 0.001), and internalizing/sexual issues (*β* = 0.022, 95% CI: 0.012–0.033; *p* < 0.001).

The significant negative interaction between the elapsed time (in years) since a placement and cumulative placement count suggests that the effect of placement instability on sleep disturbance modestly attenuated over time (interaction between elapsed time and three-time placement *β* = −0.035, 95% CI: −0.067 to −0.003; *p* = 0.034; interaction between elapsed time and four-time placement *β* = −0.048, 95% CI: −0.082 to −0.014; *p* = 0.006).

## 4. Discussion

### 4.1. Primary Findings

In this large longitudinal cohort of youth involved in the child welfare system, sleep disturbance increased from baseline to the end of follow-up and placement instability exhibited a clear dose–response effect with worsening sleep disturbance over time. Descriptively, the number of youths with moderate or severe sleep disturbance increased from 2016 (9.65%) at baseline to 2884 (13.80%) by the end of follow-up, indicating that sleep problems were not only common at entry into care but became more prevalent over time. More importantly, the time-lagged model showed that youths with two, three, and four or more placements had progressively higher levels of sleep disturbance over time, relative to youth with a single, stable placement, even after adjustment for sleep at the beginning of each placement, demographic characteristics, and baseline factor scores reflecting clinical and psychosocial needs.

The primary finding aligns with the prior literature indicating that instability in caregiving environments can disrupt children’s sense of safety, predictability, and emotional regulation, all of which are important for healthy sleep patterns [8,11,21,22]. Research has shown that frequent placement changes may introduce new caregivers, environments, and expectations, potentially increasing stress and vigilance that can interfere with a child’s initiation and maintenance of restful sleep [3,8,33]. Tinienko et al. [8], for example, documented significant sleep disruption among young foster care youth, including longer sleep latency and greater variability in sleep duration, suggesting that instability and disruption in caregiving environments may be important contributors to these difficulties. Additional prior work has similarly shown that disruptions in caregiving environments are associated with sleep problems, anxiety, and caregiver strain, factors that may further compromise sleep health among youth in out-of-home care [33]. In this sense, the present findings do not merely suggest that sleep problems and placement instability coexist; rather, they support the interpretation that repeated disruptions in care environments are associated with progressively worse sleep functioning over time. Further, these findings are also consistent with developmental and trauma-informed frameworks suggesting that repeated caregiving disruption may dysregulate arousal, perceived safety, and emotional regulation, processes that are central to sleep–wake functioning in children and adolescents [24,25,26,34].

A major strength of our primary finding is that the association between placement instability and later sleep disturbance remained after accounting for sleep status at the time of placement. This is relevant as it addresses a competing explanation that youth with pre-existing sleep difficulties might simply be more likely to experience placement disruption. In the present study, sleep disturbance at placement entry showed a strong autoregressive effect on later sleep disturbance within the same placement period, indicating that sleep problems are highly persistent once present. However, even after accounting for that persistence, cumulative placement count remained positively associated with subsequent sleep disturbance. This suggests that the observed relationship is not solely explained by baseline vulnerability, but rather that placement instability is associated with additional worsening in sleep quality over time. Unlike cross-sectional or baseline-to-endpoint comparisons, the time-lagged approach allowed later sleep disturbance to be examined in relation to placement experiences, while also accounting for sleep at placement entry, thereby improving temporal ordering and strengthening confidence that the findings reflect changes occurring during foster care involvement rather than only stable differences between youth.

In addition, the elapsed time since placement was also positively associated with later sleep disturbance, while the interaction between elapsed time and higher placement counts suggested modest attenuation over time among youth with three or more placements. This pattern, however, warrants a cautious interpretation. On the one hand, the positive main effect of time suggests that sleep difficulties may continue to emerge or remain visible as placement periods evolve rather than resolve quickly after transition. The attenuated effect among higher placement counts may reflect partial adaptation within the analytic sample, or the possibility that the initial disruption associated with repeated placement is strongest earlier in the placement period. Even with this attenuation, however, the overall pattern remained as one in which greater placement instability was associated with worse subsequent sleep.

The strong persistence of sleep disturbance across placement periods is clinically meaningful [1,2,34]. It indicates that sleep problems observed at or near the beginning of a placement may not resolve spontaneously and may instead represent a stable marker of ongoing dysregulation. From a developmental perspective, this pattern is consistent with the literature showing that sleep and emotional or behavioral functioning are tightly intertwined over time, such that disruptions in one domain may reinforce difficulties in the other [1,2,34]. In this context, sleep disturbance may function both as an indicator of stress and as a mechanism through which instability contributes to poor adjustment. Taken together, these findings suggest two related processes: first, sleep problems tend to persist once present in youth; second, repeated placement instability is associated with incremental worsening of sleep beyond its initial status.

The effect of placement instability on sleep disturbance may also operate through broader behavioral, emotional, and health-related pathways. Prior research has shown that placement instability is associated with increased risk of behavioral difficulties, internalizing symptoms, and trauma-related distress among youth in foster care. A recent systematic review by Maguire et al. (2024) concluded that placement instability is a consistent predictor of worse behavioral and mental health outcomes in foster youth, particularly externalizing behavior problems [13]. Other studies have linked instability to internalizing problems and trauma-related symptoms, each of which can interfere with sleep [15,29,35,36]. The evidence suggests that this relationship may be bidirectional: instability may worsen behavioral and emotional health, while children’s pre-existing behavioral issues may also contribute to placement disruption [28,36,37]. This feedback loop, in which behavioral challenges increase the risk of moves and repeated moves, highlights the importance of considering sleep within the broader context of youth functioning and placement experiences in out-of-home care.

Overall, our findings suggest that sleep disturbance is not only prevalent among youth in foster care, but also sensitive to the cumulative burden of placement instability. The present study extends prior work by showing, within a longitudinal framework, that repeated placement instability is associated with progressively worse later onset sleep disturbance even after accounting for sleep at placement entry and measured psychosocial needs. These findings position sleep disturbance as both a meaningful outcome and a potentially useful marker of adjustment during foster care transitions.

### 4.2. Secondary Findings

Beyond placement moves, this study also found that placement settings and their restrictiveness affect sleep outcomes. Youth who spent more time in kinship care tended to experience lower subsequent sleep disturbance, whereas time spent in treatment foster homes was associated with higher levels of sleep disturbance. These differences may reflect variation in stability, routines, familiarity with caregivers, and perceived safety across placement settings. Kinship care placements may provide greater continuity of relationships and cultural or familial familiarity, which could support emotional regulation and sleep. These findings are broadly consistent with prior works showing that kinship care is often associated with greater stability and better behavioral outcomes than non-kin foster placements [38,39,40,41,42], while multiple moves and higher restrictiveness elevate risk for behavioral and emotional problems that may in turn undermine sleep [13,14,20,36]. For example, Rubin et al. (2008) found that children placed with relatives had significantly lower behavioral problem scores after 3 years than children placed in non-kin foster homes [43]. In that study, the predicted probability of behavioral problems at 36 months was 32% for children in kinship care compared with 46% for non-kin foster care, even after adjusting for baseline risk and placement stability [43]. Children in kinship care were also significantly less likely to experience unstable placements than children in foster care [43]. A stable placement with familiar relatives may foster a greater sense of comfort, continuity, and emotional security, which could reduce nighttime vigilance and anxiety, and in turn, support healthier sleep in youth.

Placement in treatment foster homes for this cohort also warrants attention. Youth spending more time in treatment foster care had higher subsequent sleep disturbance, even after accounting for baseline sleep and other baseline factors (Table 2). One possible explanation is that treatment foster care often serves youth with more complex emotional, behavioral, or trauma-related needs, such that these settings may reflect the concentration of higher-risk children rather than a purely setting-driven effect. At the same time, the results highlight that sleep disturbance remains an important unresolved issue even within settings designed to provide more intensive support.

### 4.3. Tertiary Findings

Baseline psychosocial and health characteristics were also important predictors of sleep disturbance. Youth with higher baseline scores related to trauma in the family, medical and developmental needs, and internalizing difficulties experienced higher subsequent sleep disturbance during out-of-home care. These findings suggest that sleep disturbance may reflect broader vulnerability related to trauma exposure, mental health concerns, and developmental challenges. Sleep should therefore be considered within the context of the broader clinical and psychosocial needs of youth involved in the child welfare system.

These findings are also conceptually consistent with developmental and trauma-informed frameworks. Early adversity and caregiving disruption have been linked to dysregulation of stress-response systems, emotional reactivity, and difficulties with sleep–wake regulation [13,22,34]. Likewise, sleep disturbance is often closely intertwined with internalizing symptoms, trauma responses, and daytime functioning in both community and high-risk populations [5,29,34,36]. In this context, it is not surprising that youth entering care with more pronounced trauma-related, medical/developmental, and internalizing vulnerabilities were more likely to exhibit later sleep disturbance. Rather than representing an isolated symptom, sleep may capture the cumulative expression of multiple forms of vulnerability that shape adaptation and adjustment to environments during foster care placement and involvement.

Descriptive findings also revealed meaningful demographic disparities. Youth with higher placement counts tended to enter care at older ages, experience shorter individual placements, and spend longer total time in care. Racial disparities were also evident across placement instability groups and sleep outcomes. Black youth were disproportionately represented among those experiencing the highest numbers of placements and among those with more severe sleep disturbance by the end of follow-up. These patterns likely reflect broader structural and systemic inequities within child welfare systems rather than individual-level differences. Addressing these disparities will require continued attention to equitable service provision, placement practices, and supportive services.

The racial disparities observed in this study are especially important because they suggest that sleep disturbance and placement instability are embedded within broader structural patterns of inequity. Differential surveillance, access to services, placement opportunities, and support resources may all shape how children move through foster care systems and the kinds of burdens they carry over time. While the present study was not designed to identify the mechanisms underlying these disparities, the findings underscore the importance of interpreting the findings within a structural rather than individualized framework.

Age-related patterns also emerged in the cohort. Younger children in our cohort exhibited slightly lower baseline sleep disturbance but demonstrated steeper increase over time, whereas older adolescents began with somewhat higher baseline sleep concerns but experienced a more modest increase across follow-up (Table 3 and Table 4). These patterns may reflect developmental differences in how youth respond to placement disruption. Prior literature has suggested that older youth in foster care may benefit from greater autonomy, more developed coping strategies, or stronger access to supportive relationships, all of which may help buffer the effects of instability on functional outcomes [30,44,45]. In this context, older adolescents may be drawing on accumulated adaptive capacities that buffer the impact of placement instability on sleep-related functioning. In contrast, younger children may be more vulnerable to placement-related disruptions because they rely more heavily on caregiver-mediated regulation, consistent routines, and stable attachment relationships [1,24,26,27].

Sleep in younger children is often more dependent on environmental predictability and caregiver support, which may make them especially sensitive to repeated changes in care settings and caregiving context [1,26,27]. These developmental differences warrant further investigation in future research, particularly in studies designed to evaluate whether age modifies the effects of placement instability and placement setting on sleep outcomes.

### 4.4. Strengths and Limitations

This study has several important strengths. First, it used a longitudinal design with substantial follow-up after entry into foster care and relied on systematic CANS assessment data collected at regular intervals by trained caseworkers. By following the same cohort over time, we were able to examine how placement experiences were associated with later sleep disturbance and improve temporal ordering between exposure and outcome.

In addition, we adjusted for baseline sleep disturbance, demographic characteristics, and a broad set of baseline functional- and clinical-need domains, which strengthens confidence that the observed associations were not solely explained by measured pre-existing differences. In the time-lagged linear mixed-effects models, placement instability remained associated with higher subsequent sleep disturbance even after adjusting for sleep status at the time of placement, time since placement, baseline trait profiles, and demographics. Because this modeling strategy evaluates sleep after the start of each placement while accounting for sleep at placement entry, it strengthens temporal ordering and supports the interpretation that changes in placement experiences are causally associated with changes in sleep disturbance within the same youth over time. Another strength is the focus on sleep disturbance as a practical and clinically relevant outcome in a large sample of youth involved in out-of-home care, which is a population often underrepresented in longitudinal health research.

However, our study has some limitations. First, although the time-lagged linear mixed-effects approach reduces confounding by time-invariant child characteristics, residual time-varying confounding remains possible, including changes in crisis severity and caregiver capacity that may influence both placement pathways and sleep. Second, CANS ratings may reflect caseworker synthesis of information from multiple sources; while interrater reliability has been demonstrated in prior validation work, state-specific interrater reliability estimates during the study period were not available in the administrative dataset. Variation in raters and information access across placement settings may therefore introduce measurement error.

Third, the study was conducted within a single state or region, and child welfare systems differ in their policies, service environments, and available supports. Factors such as local placement practices, resource availability, and trauma-informed caregiver training may shape both placement experiences and youth well-being. As a result, these findings may not fully generalize to other states or international settings. Replication in other jurisdictions, and ideally in multi-state datasets, would help clarify the consistency of these patterns across systems.

Finally, descriptive findings showed substantial demographic and placement-duration differences across placement settings and instability strata, including racial disparities in placement instability and sleep outcomes. These patterns underscore the importance of equity-informed interpretation and caution in attributing differences to placement setting alone.

### 4.5. Policy and Clinical Implications and Future Research

Placement stability is widely recognized as fundamental to youth well-being, given its links to safety, permanency, and developmental outcomes. In contrast, instability has been associated with elevated risk across multiple domains, including social development, academic performance, and emotional well-being [14,43,46,47]. Consistent with this broader literature, our results suggest that placement instability is associated with worsening sleep disturbance over time, highlighting sleep as a practical indicator of stress and dysregulation experienced during foster care transitions.

From a practice perspective, agencies could consider implementing standardized sleep screening at each placement change, using routinely collected tools (e.g., the CANS sleep item) supplemented by brief follow-up checks approximately 30–60 days after a move, to detect emerging sleep concerns early on. In addition, when sleep concerns are identified, or when youth experience repeated placement changes, caseworkers and clinicians could support caregiver coaching and transition planning focused on maintaining consistent bedtime routines and trauma-informed nighttime strategies (e.g., predictable evening schedules, calming pre-sleep routines, and reinforcement of safety cues), paired with clear referral pathways to pediatric or behavioral health services for evidence-informed sleep interventions when actionable sleep disturbance persists.

Future research is needed to clarify the mechanisms and sequencing of sleep and sleep disruption amongst youth in out-of-home care. Sleep disruption related to placement transitions may contribute to downstream challenges associated with instability, including behavioral and emotional dysregulation and impaired functioning. In the present study, we adjusted for baseline functioning and co-occurring needs to isolate the association between placement instability and sleep disturbance; however, future work should examine these processes longitudinally to better characterize directionality and timing. For example, research using repeated assessments across placement episodes could test whether sleep changes precede subsequent changes in functioning or behavioral health needs and whether targeted supports during transitions reduce sleep deterioration among youth experiencing multiple moves.

Finally, these findings reinforce the importance of cross-sector coordination. Effective collaboration among child welfare, healthcare, mental health, and education professionals is likely necessary to develop feasible sleep-focused screening and referral workflows and to ensure that foster care youth have timely access to supportive services that promote sleep health.

## Figures and Tables

**Table 1:** Population characteristics by total placement count.

	All Children (N = 20,888)	1 (Stable) (n = 10,371)	2 (n = 5248)	3 (n = 2576)	4 (n = 1201)	5–6 (n = 950)	7–22 (n = 542)	Test Stats (*p*-Value)
**Entry Age (Years)**								
Mean (SD)	11.2 (4.04)	11.3 (4.21)	11.2 (4.03)	11.0 (3.89)	11.2 (3.72)	11.4 (3.43)	11.1 (2.98)	F(5,N-6) = 4.2
Median [Min, Max]	11.0 [5.00, 18.0]	11.0 [5.00, 18.0]	11.0 [5.00, 18.0]	11.0 [5.00, 18.0]	12.0 [5.00, 18.0]	12.0 [5.00, 17.0]	12.0 [5.00, 17.0]	(*p* < 0.001)
**Entry Age (Group)**								
5–10	9353(44.8%)	4656 (44.9%)	2423 (46.2%)	1193 (46.3%)	514 (42.8%)	360 (37.9%)	207 (38.2%)	χ^2^(10) = 488
11–15	7379 (35.3%)	3271 (31.5%)	1783 (34.0%)	990 (38.4%)	530 (44.1%)	491 (51.7%)	314 (57.9%)	(*p* < 0.001)
16–18	4156 (19.9%)	2444 (23.6%)	1042 (19.9%)	393 (15.3%)	157 (13.1%)	99 (10.4%)	21 (3.9%)	
**Gender**								
Male	11,109 (53.2%)	5614 (54.1%)	2777 (52.9%)	1363 (52.9%)	637 (53.0%)	454 (47.8%)	264 (48.7%)	χ^2^(5) = 19.4
Female	9779 (46.8%)	4757 (45.9%)	2471 (47.1%)	1213 (47.1%)	564 (47.0%)	496 (52.2%)	278 (51.3%)	(*p* = 0.002)
**Race**								
N/A	287 (1.4%)	184 (1.8%)	55 (1.0%)	23 (0.9%)	19 (1.6%)	5 (0.5%)	1 (0.2%)	
White	12,146 (58.1%)	6372 (61.4%)	3081 (58.7%)	1421 (55.2%)	646 (53.8%)	426 (44.8%)	200 (36.9%)	
Black	6815 (32.6%)	2964 (28.6%)	1705 (32.5%)	946 (36.7%)	446 (37.1%)	445 (46.8%)	309 (57.0%)	Fisher Exact
Asian	281 (1.3%)	152 (1.5%)	81 (1.5%)	31 (1.2%)	10 (0.8%)	6 (0.6%)	1 (0.2%)	(*p* < 0.001)
American Indian/Alaska Native	1321 (6.3%)	671 (6.5%)	321 (6.1%)	151 (5.9%)	80 (6.7%)	68 (7.2%)	30 (5.5%)	
Native Hawaiian/Pacific Islander	38 (0.2%)	28 (0.3%)	5 (0.1%)	4 (0.2%)	0 (0%)	0 (0%)	1 (0.2%)	
**Ethnicity**								
N/A	1248 (6.0%)	703 (6.8%)	286 (5.4%)	117 (4.5%)	66 (5.5%)	50 (5.3%)	26 (4.8%)	χ^2^(10) = 37.6
Non–Hispanic	17,419 (83.4%)	8586 (82.8%)	4376 (83.4%)	2210 (85.8%)	990 (82.4%)	788 (82.9%)	469 (86.5%)	(*p* < 0.001)
Hispanic	2221 (10.6%)	1082 (10.4%)	586 (11.2%)	249 (9.7%)	145 (12.1%)	112 (11.8%)	47 (8.7%)	
**Avg. Length of Placement (Years)**								
Mean (SD)	0.957 (0.837)	0.956 (0.938)	0.979 (0.765)	0.999 (0.745)	0.920 (0.655)	0.874 (0.589)	0.767 (0.463)	F(5,N-6) = 10.0
Median [Min, Max]	0.73 [0.08, 9.43]	0.69 [0.08, 9.43]	0.79 [0.09, 7.30]	0.83 [0.10, 6.03]	0.75 [0.10, 6.19]	0.72 [0.12, 5.12]	0.65 [0.11, 3.64]	(*p* < 0.001)
**Avg. Length of Placement (Group)**								
30–183 days	7125 (34.1%)	4113 (39.7%)	1556 (29.6%)	666 (25.9%)	353 (29.4%)	260 (27.4%)	177 (32.7%)	
184–365 days	6484 (31.0%)	2738 (26.4%)	1744 (33.2%)	915 (35.5%)	451 (37.6%)	395 (41.6%)	241 (44.5%)	Fisher Exact
366–730 days	5304 (25.4%)	2400 (23.1%)	1464 (27.9%)	765 (29.7%)	314 (26.1%)	250 (26.3%)	111 (20.5%)	(*p* < 0.001)
731–1095 days	1345 (6.4%)	700 (6.7%)	353 (6.7%)	172 (6.7%)	71 (5.9%)	37 (3.9%)	12 (2.2%)	
1096–3445 days	630 (3.0%)	420 (4.0%)	131 (2.5%)	58 (2.3%)	12 (1.0%)	8 (0.8%)	1 (0.2%)	
**Total Length of Placement (Years)**								
Mean (SD)	1.75 (1.62)	0.956 (0.938)	1.78 (1.28)	2.59 (1.51)	3.21 (1.68)	4.01 (1.78)	5.41 (1.99)	F(5,N-6) = 2994
Median [Min, Max]	1.25 [0.08, 9.84]	0.68 [0.08, 9.43]	1.45 [0.18, 9.50]	2.27 [0.38, 9.59]	2.87 [0.47, 9.84]	3.71 [0.84, 9.76]	5.19 [1.58, 9.80]	(*p* < 0.001)
**Total Length of Placement (Group)**								
30–183 days	4538 (21.7%)	4113 (39.7%)	403 (7.7%)	20 (0.8%)	2 (0.2%)	0 (0%)	0 (0%)	
184–365 days	4152 (19.9%)	2738 (26.4%)	1149 (21.9%)	237 (9.2%)	25 (2.1%)	3 (0.3%)	0 (0%)	Fisher Exact
366–730 days	5668 (27.1%)	2400 (23.1%)	2039 (38.9%)	821 (31.9%)	299 (24.9%)	103 (10.8%)	6 (1.1%)	(*p* < 0.001)
731–1095 days	2923 (14.0%)	700 (6.7%)	947 (18.0%)	694 (26.9%)	319 (26.6%)	217 (22.8%)	46 (8.5%)	
1096–3595 days	3607 (17.3%)	420 (4.0%)	710 (13.5%)	804 (31.2%)	556 (46.3%)	627 (66.0%)	490 (90.4%)	

Note: The missing value “N/A” in race and ethnicity represents “refused to report or unknown”; when conducting statistical analysis with categorical variables, the reference levels are: 5–10 for age, male for gender, N/A for race, and N/A for ethnicity.

**Table 2:** Population characteristics by primary placement types.

	All Children (N = 20,888)	Missing (n = 96)	Exit (n = 98)	Kinship (n = 3229)	Foster (n = 10,795)	TFC (n = 2652)	Congregate (n = 3808)	Institute (n = 210)	Test Stats (*p*-Value)
**Entry Age (Years)**									
Mean (SD)	11.2 (4.04)	16.0 (1.19)	14.1 (4.56)	9.98 (3.55)	10.2 (3.94)	11.3 (4.04)	14.7 (2.20)	15.4 (1.65)	F(6,N-7) = 859
Median [Min, Max]	11.0 [5.00, 18.0]	16.0 [13.0, 18.0]	16.0 [5.00, 18.0]	9.00 [5.00, 18.0]	10.0 [5.00, 18.0]	12.0 [5.00, 18.0]	15.0 [5.00, 18.0]	16.0 [5.00, 18.0]	(*p* < 0.001)
**Entry Age (Group)**									
5–10	9353 (44.8%)	0 (0.0%)	20 (20.4%)	1908 (59.1%)	6052 (56.1%)	1145 (43.2%)	224 (5.9%)	4 (1.9%)	Fisher Exact
11–15	7379 (35.3%)	32 (33.3%)	16 (16.3%)	1017 (31.5%)	3239 (30.0%)	967 (36.5%)	2019 (53.0%)	89 (42.4%)	(*p* < 0.001)
16–18	4156 (19.9%)	64 (66.7%)	62 (63.3%)	304 (9.4%)	1504 (13.9%)	540 (20.4%)	1565 (41.1%)	117 (55.7%)	
**Gender**									
Male	11,109 (53.2%)	27 (28.1%)	31 (31.6%)	1572 (48.7%)	5414 (50.2%)	1490 (56.2%)	2400 (63.0%)	175 (83.3%)	χ^2^(6) = 343.0
Female	9779 (46.8%)	69 (71.9%)	67 (68.4%)	1657 (51.3%)	5381 (49.8%)	1162 (43.8%)	1408 (37.0%)	35 (16.7%)	(*p* < 0.001)
**Race**									
N/A	287 (1.4%)	2 (2.1%)	2 (2.0%)	33 (1.0%)	147 (1.4%)	24 (0.9%)	72 (1.9%)	7 (3.3%)	
White	12,146 (58.1%)	28 (29.2%)	36 (36.7%)	1891 (58.6%)	6738 (62.4%)	1356 (51.1%)	2008 (52.7%)	89 (42.4%)	
Black	6815 (32.6%)	62 (64.6%)	57 (58.2%)	1011 (31.3%)	3001 (27.8%)	1102 (41.6%)	1477 (38.8%)	105 (50.0%)	Fisher Exact
Asian	281 (1.3%)	1 (1.0%)	1 (1.0%)	57 (1.8%)	159 (1.5%)	26 (1.0%)	36 (0.9%)	1 (0.5%)	(*p* < 0.001)
American Indian/Alaska Native	1321 (6.3%)	3 (3.1%)	2 (2.0%)	235 (7.3%)	727 (6.7%)	142 (5.4%)	204 (5.4%)	8 (3.8%)	
Native Hawaiian/Pacific Islander	38 (0.2%)	0 (0.0%)	0 (0.0%)	2 (0.1%)	23 (0.2%)	2 (0.1%)	11 (0.3%)	0 (0.0%)	
**Ethnicity**									
N/A	1248 (6.0%)	13 (13.5%)	12 (12.2%)	119 (3.7%)	546 (5.1%)	175 (6.6%)	332 (8.7%)	51 (24.3%)	Fisher Exact
Non–Hispanic	17,419 (83.4%)	67 (69.8%)	82 (83.7%)	2776 (86.0%)	9018 (83.5%)	2189 (82.5%)	3147 (82.6%)	140 (66.7%)	(*p* < 0.001)
Hispanic	2221 (10.6%)	16 (16.7%)	4 (4.1%)	334 (10.3%)	1231 (11.4%)	288 (10.9%)	329 (8.6%)	19 (9.0%)	
**Avg. Length of Placement (Years)**									
Mean (SD)	0.957 (0.837)	0.474 (0.399)	0.572 (0.410)	0.869 (0.566)	1.03 (0.862)	1.31 (1.11)	0.641 (0.596)	0.408 (0.227)	F(6,N-7) = 224
Median [Min, Max]	0.73 [0.08, 9.43]	0.38 [0.09, 3.19]	0.46 [0.09, 2.34]	0.79 [0.08, 6.27]	0.81 [0.08, 9.16]	1.00 [0.08, 9.43]	0.49 [0.08, 7.43]	0.37 [0.08, 1.40]	(*p* < 0.001)
**Avg. Length of Placement (Group)**									
30–183 days	7125 (34.1%)	57 (59.4%)	55 (56.1%)	967 (29.9%)	3340 (30.9%)	586 (22.1%)	1960 (51.5%)	160 (76.2%)	
184–365 days	6484 (31.0%)	33 (34.4%)	28 (28.6%)	1191 (36.9%)	3178 (29.4%)	747 (28.2%)	1260 (33.1%)	47 (22.4%)	Fisher Exact
366–730 days	5304 (25.4%)	5 (5.2%)	14 (14.3%)	963 (29.8%)	3037 (28.1%)	813 (30.7%)	469 (12.3%)	3 (1.4%)	(*p* < 0.001)
731–1095 days	1345 (6.4%)	0 (0.0%)	1 (1.0%)	86 (2.7%)	878 (8.1%)	301 (11.4%)	79 (2.1%)	0 (0.0%)	
1096–3445 days	630 (3.0%)	1 (1.0%)	0 (0.0%)	22 (0.7%)	362 (3.4%)	205 (7.7%)	40 (1.1%)	0 (0.0%)	
**Total Length of Placement (Years)**									
Mean (SD)	1.75 (1.62)	1.37 (1.03)	1.40 (1.22)	1.37 (1.16)	1.71 (1.55)	2.52 (2.00)	1.69 (1.72)	1.08 (1.36)	F(6,N-7) = 145
Median [Min, Max]	1.25 [0.08, 9.84]	1.10 [0.09, 4.05]	1.10 [0.09, 6.35]	1.10 [0.08, 9.51]	1.26 [0.08, 9.78]	2.01 [0.08, 9.84]	1.05 [0.08, 9.80]	0.44 [0.08, 8.57]	(*p* < 0.001)
**Total Length of Placement (Group)**									
30–183 days	4538 (21.7%)	20 (20.8%)	29 (29.6%)	634 (19.6%)	2418 (22.4%)	306 (11.5%)	1016 (26.7%)	115 (54.8%)	
184–365 days	4152 (19.9%)	22 (22.9%)	18 (18.4%)	835 (25.9%)	2045 (18.9%)	372 (14.0%)	829 (21.8%)	31 (14.8%)	χ^2^(24) = 1205
366–730 days	5668 (27.1%)	29 (30.2%)	28 (28.6%)	1166 (36.1%)	2933 (27.2%)	640 (24.1%)	845 (22.2%)	27 (12.9%)	(*p* < 0.001)
731–1095 days	2923 (14.0%)	16 (16.7%)	16 (16.3%)	355 (11.0%)	1617 (15.0%)	473 (17.8%)	425 (11.2%)	21 (10.0%)	
1096–3595 days	3607 (17.3%)	9 (9.4%)	7 (7.1%)	239 (7.4%)	1782 (16.5%)	861 (32.5%)	693 (18.2%)	16 (7.6%)	

Table 2 summarizes demographic and placement-duration characteristics for 20,888 children stratified by primary placement type. Across placement types, differences were statistically significant for age, gender, race, ethnicity, and placement-duration measures (all *p* < 0.001). Demographic characteristics: Entry age differed markedly by primary placement type (*p* < 0.001). Children in kinship and foster care entered at younger ages (mean 9.98 years and 10.2 years, respectively), whereas youth in congregate and institutional care entered at substantially older ages (mean 14.7 years and 15.4 years, respectively). Consistent with this pattern, the proportion of children entering care at ages 5–10 was highest for kinship (59.1%) and foster care (56.1%) but was much lower for congregate (5.9%) and institutional care (1.9%). Gender distributions differed significantly by placement type (*p* < 0.001). The overall sample was 53.2% male, but males comprised a larger share of youth in congregate (63.0%) and institutional care (83.3%), while the exit category was predominantly female (68.4%). Placement duration and time in care: Average placement length differed significantly by primary placement type (*p* < 0.001). The mean average length of placement was longest in TFC (1.31 years, SD 1.11) and foster care (1.03 years, SD 0.862), and shortest in institutional care (0.408 years, SD 0.227) and congregate care (0.641 years, SD 0.596).

**Table 3:** Population characteristics by sleep status—baseline.

	All Children (N = 20,888)	0 (n = 15,178)	1 (n = 3694)	2 (n = 1701)	3 (n = 315)	Test Stats (*p*-Value)
**Entry Age (Years)**						
Mean (SD)	11.2 (4.04)	11.2 (4.02)	11.1 (4.08)	11.8 (4.06)	11.2 (4.12)	F(3,N-4) = 14.6
Median [Min, Max]	11.0 [5.00, 18.0]	11.0 [5.00, 18.0]	11.0 [5.00, 18.0]	13.0 [5.00, 18.0]	11.0 [5.00, 18.0]	(*p* < 0.001)
**Entry Age (Group)**						
5–10	9353 (44.8%)	6854 (45.2%)	1704 (46.1%)	661 (38.9%)	134 (42.5%)	χ^2^(6) = 43.7
11–15	7379 (35.3%)	5371 (35.4%)	1282 (34.7%)	610 (35.9%)	116 (36.8%)	(*p* < 0.001)
16–18	4156 (19.9%)	2953 (19.5%)	708 (19.2%)	430 (25.3%)	65 (20.6%)	
**Gender**						
Male	11,109 (53.2%)	8014 (52.8%)	1978 (53.5%)	948 (55.7%)	169 (53.7%)	χ^2^(3) = 5.6
Female	9779 (46.8%)	7164 (47.2%)	1716 (46.5%)	753 (44.3%)	146 (46.3%)	(*p* = 0.135)
**Race**						
N/A	287 (1.4%)	201 (1.3%)	61 (1.7%)	21 (1.2%)	4 (1.3%)	
White	12,146 (58.1%)	8677 (57.2%)	2300 (62.3%)	993 (58.4%)	176 (55.9%)	
Black	6815 (32.6%)	5066 (33.4%)	1074 (29.1%)	562 (33.0%)	113 (35.9%)	Fisher Exact
Asian	281 (1.3%)	231 (1.5%)	32 (0.9%)	16 (0.9%)	2 (0.6%)	(*p* < 0.001)
American Indian/Alaska Native	1321 (6.3%)	982 (6.5%)	213 (5.8%)	106 (6.2%)	20 (6.3%)	
Native Hawaiian/Pacific Islander	38 (0.2%)	21 (0.1%)	14 (0.4%)	3 (0.2%)	0 (0%)	
**Ethnicity**						
N/A	1248 (6.0%)	914 (6.0%)	201 (5.4%)	122 (7.2%)	11 (3.5%)	χ^2^(6) = 16.6
Non–Hispanic	17,419 (83.4%)	12,604 (83.0%)	3127 (84.7%)	1410 (82.9%)	278 (88.3%)	(*p* = 0.011)
Hispanic	2221 (10.6%)	1660 (10.9%)	366 (9.9%)	169 (9.9%)	26 (8.3%)	
**Avg. Length of Placement (Years)**						
Mean (SD)	0.957 (0.837)	0.936 (0.811)	1.00 (0.865)	1.00 (0.927)	1.18 (1.12)	F(3,N-4) = 16.1
Median [Min, Max]	0.73 [0.08, 9.43]	0.73 [0.08, 9.43]	0.77 [0.08, 9.11]	0.73 [0.08, 7.43]	0.88 [0.08, 8.16]	(*p* < 0.001)
**Avg. Length of Placement (Group)**						
30–183 days	7125 (34.1%)	5268 (34.7%)	1176 (31.8%)	583 (34.3%)	98 (31.1%)	
184–365 days	6484 (31.0%)	4719 (31.1%)	1146 (31.0%)	533 (31.3%)	86 (27.3%)	χ^2^(12) = 64.8
366–730 days	5304 (25.4%)	3867 (25.5%)	978 (26.5%)	383 (22.5%)	76 (24.1%)	(*p* < 0.001)
731–1095 days	1345 (6.4%)	903 (5.9%)	273 (7.4%)	134 (7.9%)	35 (11.1%)	
1096–3445 days	630 (3.0%)	421 (2.8%)	121 (3.3%)	68 (4.0%)	20 (6.3%)	
**Total Length of Placement (Years)**						
Mean (SD)	1.75 (1.62)	1.68 (1.55)	1.87 (1.74)	1.97 (1.89)	2.29 (2.08)	F(3,N-4) = 38.9
Median [Min, Max]	1.25 [0.08, 9.84]	1.22 [0.08, 9.78]	1.29 [0.08, 9.84]	1.34 [0.08, 9.80]	1.77 [0.08, 9.59]	(*p* < 0.001)
**Total Length of Placement (Group)**						
30–183 days	4538 (21.7%)	3401 (22.4%)	744 (20.1%)	337 (19.8%)	56 (17.8%)	
184–365 days	4152 (19.9%)	3029 (20.0%)	733 (19.8%)	339 (19.9%)	51 (16.2%)	χ^2^(12) = 93.8
366–730 days	5668 (27.1%)	4216 (27.8%)	973 (26.3%)	411 (24.2%)	68 (21.6%)	(*p* < 0.001)
731–1095 days	2923 (14.0%)	2108 (13.9%)	501 (13.6%)	253 (14.9%)	61 (19.4%)	
1096–3595 days	3607 (17.3%)	2424 (16.0%)	743 (20.1%)	361 (21.2%)	79 (25.1%)	

Note: Sleep status is defined by the 4-level CANS item “Life Functioning/Sleep” with scores of 0–3. A rating of 0 indicates there is no evidence of a specialized need (no evidence of problems with the sleep process. The child gets a full night’s sleep each night); a rating of **1** indicates there is a history or concern that a basic special need may exist (the child has some problems sleeping and generally gets a full night’s sleep, but problems may arise once a week. This may include occasionally awakening or bed-wetting or having nightmares); a rating of **2** indicates there is the presence of a moderate special need (the child is having problems with sleep. Their sleep is often disrupted, and the child seldom obtains a full night of sleep); a rating of **3** indicates there is the presence of an intensive special need that is either dangerous or disabling (the child is sleep-deprived. Sleeping is almost always difficult, and the child is not able to obtain a full night’s sleep).

**Table 4:** Population characteristics by sleep status—end of follow-up.

	All Children (N = 20,888)	0 (n = 13,450)	1 (n = 4554)	2 (n = 2372)	3 (n = 512)	Test Stats (*p*-Value)
**Entry Age (Years)**						
Mean (SD)	11.2 (4.04)	11.4 (4.02)	11.0 (4.03)	11.0 (4.06)	10.1 (4.11)	F(3,N-4) = 28.6
Median [Min, Max]	11.0 [5.00, 18.0]	12.0 [5.00, 18.0]	11.0 [5.00, 18.0]	11.0 [5.00, 18.0]	9.00 [5.00, 18.0]	(*p* < 0.001)
**Entry Age (Group)**						
5–10	9353 (44.8%)	5833 (43.4%)	2117 (46.5%)	1119 (47.2%)	284 (55.5%)	χ^2^(6) = 59.6
11–15	7379 (35.3%)	4783 (35.6%)	1624 (35.7%)	816 (34.4%)	156 (30.5%)	(*p* < 0.001)
16–18	4156 (19.9%)	2834 (21.1%)	813 (17.9%)	437 (18.4%)	72 (14.1%)	
**Gender**						
Male	11,109 (53.2%)	7145 (53.1%)	2415 (53.0%)	1276 (53.8%)	273 (53.3%)	χ^2^(3) = 0.4
Female	9779 (46.8%)	6305 (46.9%)	2139 (47.0%)	1096 (46.2%)	239 (46.7%)	(*p* = 0.936)
**Race**						
N/A	287 (1.4%)	183 (1.4%)	72 (1.6%)	26 (1.1%)	6 (1.2%)	
White	12,146 (58.1%)	7796 (58.0%)	2800 (61.5%)	1305 (55.0%)	245 (47.9%)	
Black	6815 (32.6%)	4350 (32.3%)	1368 (30.0%)	863 (36.4%)	234 (45.7%)	Fisher Exact
Asian	281 (1.3%)	214 (1.6%)	38 (0.8%)	23 (1.0%)	6 (1.2%)	(*p* < 0.001)
American Indian/Alaska Native	1321 (6.3%)	885 (6.6%)	266 (5.8%)	150 (6.3%)	20 (3.9%)	
Native Hawaiian/Pacific Islander	38 (0.2%)	22 (0.2%)	10 (0.2%)	5 (0.2%)	1 (0.2%)	
**Ethnicity**						
N/A	1248 (6.0%)	838 (6.2%)	261 (5.7%)	131 (5.5%)	18 (3.5%)	χ^2^(6) = 10.2
Non–Hispanic	17,419 (83.4%)	11,165 (83.0%)	3828 (84.1%)	1992 (84.0%)	434 (84.8%)	(*p* = 0.117)
Hispanic	2221 (10.6%)	1447 (10.8%)	465 (10.2%)	249 (10.5%)	60 (11.7%)	
**Avg. Length of Placement (Years)**						
Mean (SD)	0.957 (0.837)	0.885 (0.761)	1.02 (0.858)	1.15 (1.03)	1.38 (1.17)	F(3,N-4) = 128
Median [Min, Max]	0.73 [0.08, 9.43]	0.70 [0.08, 9.43]	0.79 [0.08, 8.00]	0.85 [0.08, 9.11]	1.04 [0.08, 8.16]	(*p* < 0.001)
**Avg. Length of Placement (Group)**						
30–183 days	7125 (34.1%)	4929 (36.6%)	1365 (30.0%)	705 (29.7%)	126 (24.6%)	
184–365 days	6484 (31.0%)	4230 (31.4%)	1473 (32.3%)	658 (27.7%)	123 (24.0%)	χ^2^(12) = 381
366–730 days	5304 (25.4%)	3295 (24.5%)	1213 (26.6%)	649 (27.4%)	147 (28.7%)	(*p* < 0.001)
731–1095 days	1345 (6.4%)	716 (5.3%)	338 (7.4%)	221 (9.3%)	70 (13.7%)	
1096–3445 days	630 (3.0%)	280 (2.1%)	165 (3.6%)	139 (5.9%)	46 (9.0%)	
**Total Length of Placement (Years)**						
Mean (SD)	1.75 (1.62)	1.51 (1.42)	2.02 (1.75)	2.34 (1.98)	2.73 (2.08)	F(3,N-4) = 316
Median [Min, Max]	1.25 [0.08, 9.84]	1.09 [0.08, 9.69]	1.49 [0.08, 9.78]	1.79 [0.08, 9.84]	2.30 [0.08, 9.35]	(*p* < 0.001)
**Total Length of Placement (Group)**						
30–183 days	4538 (21.7%)	3359 (25.0%)	758 (16.6%)	355 (15.0%)	66 (12.9%)	
184–365 days	4152 (19.9%)	2873 (21.4%)	836 (18.4%)	381 (16.1%)	62 (12.1%)	χ^2^(12) = 875
366–730 days	5668 (27.1%)	3783 (28.1%)	1226 (26.9%)	556 (23.4%)	103 (20.1%)	(*p* < 0.001)
731–1095 days	2923 (14.0%)	1759 (13.1%)	687 (15.1%)	388 (16.4%)	89 (17.4%)	
1096–3595 days	3607 (17.3%)	1676 (12.5%)	1047 (23.0%)	692 (29.2%)	192 (37.5%)	

Note: Sleep status is defined by the 4-level CANS item “Life Functioning/Sleep” with scores of 0–3. A rating of 0 indicates there is no evidence of a specialized need (no evidence of problems with the sleep process. The child gets a full night’s sleep each night); a rating of 1 indicates there is a history or concern that a basic special need may exist (the child has some problems sleeping and generally gets a full night’s sleep, but problems may arise once a week. This may include occasionally awakening or bed-wetting or having nightmares); a rating of 2 indicates there is the presence of a moderate special need (the child is having problems with sleep. Sleep is often disrupted, and the child seldom obtains a full night of sleep); a rating of 3 indicates there is the presence of an intensive special need that is either dangerous or disabling (the child is sleep-deprived. Sleeping is almost always difficult, and the child is not able to obtain a full night’s sleep).

**Table 5:** Factor loadings on 87 CANS items.

Domains & Items	UNQ	EXT	CGV	TRF	STR	MDD	CUL	INT
CTR_SEXUAL_ABUSE	0.671	−0.116	0.012	0.168	0.047	−0.019	0.025	**0.535**
CTR_PHYSICAL_ABUSE	0.777	0.110	0.005	**0.375**	−0.063	0.044	0.065	0.095
CTR_NEGLECT	0.676	−0.126	0.017	**0.506**	0.066	0.162	0.065	−0.217
CTR_EMOTIONAL_ABUSE	0.524	0.043	0.018	**0.579**	−0.015	0.000	0.075	0.174
CTR_WITNESS_TO_FAMILY_VIOLENCE	0.731	0.075	0.049	**0.501**	−0.101	0.004	0.094	−0.130
CTR_WITNESS_TO_COMMUNITY_VIOLENCE	0.694	0.385	0.042	0.317	−0.102	−0.162	0.213	−0.147
CTR_WITNESS_VICTIM_TO_CRIMINAL_ACTIVITY	0.767	0.157	0.069	**0.427**	−0.093	−0.136	0.114	−0.130
CAD_ADJUSTMENT_TO_TRAUMA	0.327	0.144	0.043	**0.508**	−0.019	0.172	0.047	0.343
CAD_TRAUMATIC_GRIEF_AND_SEPARATION	0.573	0.141	0.042	**0.423**	0.008	0.194	0.095	0.130
CAD_INTRUSIONS	0.372	0.049	0.021	0.431	−0.018	0.211	0.060	0.431
CAD_DISSOCIATION	0.494	0.145	0.017	0.303	−0.042	0.272	0.087	0.337
CAD_ATTACHMENT_DIFFICULTIES	0.442	0.225	0.017	**0.404**	0.071	0.195	0.107	0.172
CLF_FAMILY_NUCLEAR	0.478	0.237	0.006	**0.391**	0.087	−0.167	0.098	0.239
CLF_FAMILY_EXTENDED	0.547	0.239	−0.009	**0.374**	0.138	−0.084	0.147	0.098
CLF_LIVING_SITUATION	0.405	**0.539**	0.038	0.097	0.099	0.007	0.069	0.176
CLF_DEVELOPMENTAL_INTELLECTUAL	0.427	0.162	−0.019	−0.110	0.198	**0.660**	0.054	−0.037
CLF_MEDICAL	0.793	−0.094	0.014	−0.066	0.111	**0.389**	0.133	0.077
CLF_PHYSICAL	0.686	−0.111	0.028	−0.076	0.183	**0.469**	0.157	0.057
CLF_DENTAL	0.859	−0.084	0.035	0.158	0.159	0.158	0.170	−0.165
CLF_DAILY_FUNCTIONING	0.273	0.173	−0.019	0.049	0.243	**0.679**	0.091	−0.017
CLF_SOCIAL_FUNCTIONING_PEER	0.261	**0.615**	−0.011	0.028	0.129	0.232	0.071	0.118
CLF_SOCIAL_FUNCTIONING_ADULTS	0.253	**0.681**	0.030	0.095	0.099	0.126	0.059	0.080
CLF_LEGAL	0.247	0.583	−0.038	−0.224	0.179	−0.427	0.046	0.187
CLF_EATING_DISTURBANCE	0.664	−0.070	−0.024	0.135	0.106	**0.354**	0.136	0.249
CLF_SEXUAL_DEVELOPMENT	0.572	0.145	0.039	−0.017	0.072	−0.075	0.102	**0.496**
CLF_LIFE_SKILLS	0.478	0.138	−0.014	−0.090	0.407	0.352	0.127	0.094
CSC_ATTENDANCE	0.648	0.440	0.050	−0.040	0.145	−0.294	0.099	−0.031
CSC_BEHAVIOR	0.276	**0.908**	0.012	0.049	−0.015	0.095	−0.022	−0.148
CSC_ACHIEVEMENT	0.469	**0.631**	0.015	−0.002	0.184	−0.023	0.073	−0.084
CSC_RELATIONSHIPS_WITH_TEACHERS	0.270	**0.879**	0.020	0.046	0.052	−0.005	0.017	−0.145
CCF_LANGUAGE	0.560	−0.101	−0.041	−0.283	−0.069	0.252	**0.651**	−0.013
CCF_CULTURAL_IDENTITY	0.443	0.019	−0.056	0.020	−0.037	0.000	**0.715**	0.116
CCF_TRADITIONS_AND_RITUALS	0.383	−0.026	−0.003	0.009	0.019	0.011	**0.783**	−0.004
CCF_CULTURE_STRESS	0.260	0.025	0.004	−0.021	−0.080	0.001	**0.890**	−0.024
CCF_KNOWLEDGE_CONGRUENCE	0.366	−0.007	0.004	0.071	0.109	−0.039	**0.738**	−0.018
CCF_HELP_SEEKING_CONGRUENCE	0.377	−0.013	0.003	0.100	0.138	−0.053	**0.717**	−0.045
CCF_EXPRESSION_OF_DISTRESS	0.397	0.046	0.019	0.144	0.098	−0.038	**0.675**	−0.034
CBE_PSYCHOSIS	0.492	0.185	−0.050	−0.007	0.005	0.276	−0.028	**0.516**
CBE_IMPULSIVE_HYPERACTIVE	0.347	**0.653**	0.003	0.047	0.023	0.264	0.005	0.091
CBE_DEPRESSION	0.364	0.088	0.019	0.203	0.076	−0.092	0.062	**0.612**
CBE_ANXIETY	0.404	0.145	0.015	0.269	0.042	0.167	0.099	**0.443**
CBE_OPPOSITIONAL	0.203	**0.800**	0.004	0.059	−0.011	0.034	0.005	0.131
CBE_CONDUCT	0.216	**0.753**	−0.038	−0.007	0.021	−0.045	0.030	0.193
CBE_ANGER_CONTROL	0.249	**0.764**	0.012	0.064	−0.015	0.092	−0.014	0.138
CBE_SOMATIZATION	0.619	0.012	0.074	0.150	0.097	0.164	0.135	**0.374**
CBE_BEHAVIORAL_REGRESSION	0.456	0.206	0.048	0.207	0.010	**0.552**	0.060	0.070
CBE_AFFECT_DYSREGULATION	0.376	0.330	−0.006	0.138	0.090	0.329	0.101	0.260
CRS_SUICIDE_RISK	0.312	0.000	−0.005	−0.020	0.047	−0.115	0.001	**0.820**
CRS_NON_SUICIDAL_SELF_INJURIOUS_BEHAVIOR	0.407	−0.024	−0.027	−0.035	0.054	0.003	0.024	**0.763**
CRS_OTHER_SELF_HARM	0.453	0.318	−0.021	−0.045	0.044	0.048	0.106	**0.456**
CRS_EXPLOITED	0.587	−0.028	0.042	0.211	0.113	−0.064	0.235	0.377
CRS_DANGER_TO_OTHERS	0.406	**0.568**	−0.002	−0.036	−0.018	0.158	−0.004	0.280
CRS_SEXUAL_AGGRESSION	0.724	0.261	0.022	−0.077	0.040	0.045	0.037	0.308
CRS_DELINQUENT_BEHAVIOR	0.221	**0.632**	−0.034	−0.178	0.147	−0.408	0.057	0.182
CRS_RUNAWAY	0.456	0.411	0.008	−0.039	0.132	−0.407	0.044	0.248
CRS_INTENTIONAL_MISBEHAVIOR	0.302	**0.732**	0.004	0.037	0.050	−0.027	0.059	0.085
CRS_FIRE_SETTING	0.769	0.364	0.006	−0.001	0.061	0.020	−0.032	0.137
CRS_BULLYING	0.479	**0.698**	0.024	0.076	−0.015	−0.068	0.078	−0.023
CST_RELATIONSHIP_PERMANENCE	0.587	−0.076	−0.002	0.498	0.336	−0.086	0.046	0.017
CST_FAMILY_NUCLEAR	0.510	−0.041	0.017	**0.519**	0.312	−0.188	0.087	0.062
CST_FAMILY_EXTENDED	0.517	−0.001	−0.001	0.403	0.426	−0.095	0.123	0.003
CST_POSITIVE_PEER_RELATIONS	0.245	0.468	0.012	0.017	0.410	0.123	0.004	0.115
CST_OPTIMISM	0.324	0.160	0.027	0.136	0.491	−0.042	0.016	0.291
CST_DECISION_MAKING	0.221	0.419	0.019	−0.068	0.474	0.137	0.002	0.147
CST_WELL_BEING	0.238	0.148	0.020	0.029	**0.563**	0.137	−0.016	0.308
CST_EDUCATIONAL_SETTING	0.473	0.460	0.049	0.037	0.398	−0.064	0.035	−0.105
CST_RECREATIONAL	0.381	0.017	0.039	−0.017	**0.758**	−0.044	0.044	0.013
CST_VOCATIONAL	0.377	−0.046	0.006	−0.062	**0.803**	0.082	0.038	−0.029
CST_TALENTS_AND_INTERESTS	0.349	0.008	0.030	−0.006	**0.795**	−0.005	0.035	−0.023
CST_SPIRITUAL_RELIGIOUS	0.408	−0.088	0.044	0.025	**0.805**	−0.115	0.020	−0.017
CST_COMMUNITY_LIFE	0.313	−0.027	0.018	0.028	**0.795**	−0.080	0.069	0.050
CST_INVOLVEMENT_WITH_CARE	0.322	0.134	0.052	−0.023	**0.735**	0.115	0.025	−0.073
CST_NATURAL_SUPPORTS	0.381	0.010	−0.030	0.185	**0.682**	−0.054	0.102	0.009
CST_RESILIENCY	0.261	0.113	0.003	0.046	**0.698**	0.153	−0.007	0.103
CST_RESOURCEFULNESS	0.244	0.065	0.014	−0.002	**0.775**	0.180	−0.013	0.042
CCG_SUPERVISION	0.281	0.091	**0.847**	0.015	−0.025	−0.061	−0.050	0.032
CCG_PROBLEM_SOLVING	0.223	0.032	**0.880**	0.026	−0.005	0.019	−0.015	−0.033
CCG_INVOLVEMENT_WITH_CARE	0.297	0.032	**0.833**	−0.052	0.010	−0.005	0.034	0.000
CCG_KNOWLEDGE	0.366	0.039	**0.749**	0.000	0.091	0.063	0.045	0.058
CCG_EMPATHY_WITH_CHILD	0.436	0.109	**0.704**	−0.047	0.065	−0.019	0.027	0.094
CCG_ORGANIZATION	0.297	−0.014	**0.841**	−0.021	0.027	−0.021	−0.029	−0.028
CCG_SOCIAL_RESOURCES	0.362	−0.103	**0.767**	−0.029	0.203	0.013	−0.041	−0.048
CCG_MEDICAL_PHYSICAL_HEALTH	0.579	−0.080	**0.652**	0.014	−0.028	0.034	−0.073	−0.016
CCG_MENTAL_HEALTH	0.416	−0.127	**0.758**	0.066	−0.058	−0.030	−0.012	0.065
CCG_SUBSTANCE_USE	0.482	−0.040	**0.710**	−0.014	−0.073	−0.097	−0.018	−0.070
CCG_FAMILY_STRESS	0.341	0.005	**0.809**	0.060	−0.061	0.077	−0.036	−0.056
CCG_CULTURAL_CONGRUENCE	0.395	−0.026	0.594	−0.108	−0.128	0.019	0.469	0.061

The rows represent 87 CANS items organized by domains, where the domain names are given by the prefix: CTR—Traumatic Experience; CAD—Adjustment to Trauma; CLF—Life Functioning; CSC—Schooling; CCF—Cultural Factors; CBE—Behavioral and Emotional Needs; CRS—Risky Behaviors. The columns represent 7 factors: EXT—Externalizing (Behaviors); CGV—Caregivers; TRF—Trauma in Family; STR—(Child) Strengths; MDD—Medical and Developmental; CUL—Cultural; INT—Internalizing (Behaviors). The loadings are color-coded by size and direction (red for positive, blue for negative); loadings of primarily loaded items are highlighted in bold. An item is considered primarily loaded on a factor if (1) said item has the largest loading on said factor out of all 7 factors; and (2) the second largest loading of said item on other factors is at least 0.10 smaller than the largest one. The special first column “UNQ” gives the remaining uniqueness of each item not associated with the factors. A high uniqueness of an item means it does not contribute to the measurement of any latent dimension represented by the 7-factor solution but instead measures another latent construct on its own.

**Table 6:** Results of time-lagged longitudinal analysis.

Domain	Variable	Category	Remark	Beta	95%CI	95%CI	*p*-Value
Demographics	Age	06~10 *	(Years)	0.000	0.000	0.000	
		11~15	(Years)	−0.040	−0.056	−0.025	0.000
		16~18	(Years)	−0.053	−0.075	−0.030	0.000
	Gender	Male *		0.000	0.000	0.000	
		Female		0.000	−0.014	0.013	0.973
	Race	N/A *	(Refused/Unknown)	0.000	0.000	0.000	
		White		−0.039	−0.104	0.025	0.229
		Black	Black/African American	0.007	−0.058	0.072	0.833
		Asian		−0.096	−0.185	−0.007	0.034
		AI/AN	American Indian/Alaskan Native	−0.042	−0.111	0.027	0.231
		NH/PI	Native Hawaiian/Pacific Islander	−0.083	−0.245	0.078	0.312
	Ethnicity	N/A *	(Refused/Unknown)	0.000	0.000	0.000	
		NH	Non-Hispanic	0.021	−0.008	0.050	0.149
		HS	Hispanic	0.039	0.005	0.074	0.024
Baseline Traits	EXT		(Factor Score) Externalizing	0.005	−0.005	0.015	0.292
	CGV		(Factor Score) Caregiver	−0.005	−0.013	0.003	0.215
	TRF		(Factor Score) Trauma in Family	0.023	0.016	0.030	0.000
	STR		(Factor Score) Child Strengths	−0.001	−0.009	0.007	0.785
	MDD		(Factor Score) Med/Development	0.028	0.019	0.037	0.000
	CUL		(Factor Score) Cultural	−0.004	−0.014	0.005	0.352
	INT		(Factor Score) Internalizing/Sexual	0.022	0.012	0.033	0.000
Autoregressive	Sleep_0		Sleep at the Day of Placement	0.816	0.807	0.825	0.000
Placement Instability	CPC	1-time *	Cumulative Placement Count	0.000	0.000	0.000	
		2-time	Cumulative Placement Count	0.025	0.004	0.046	0.020
		3-time	Cumulative Placement Count	0.045	0.017	0.074	0.002
		4+ time	Cumulative Placement Count	0.067	0.033	0.101	0.000
Placement Types	EXC		Cumulative Years—Exiting Status	−0.089	−0.209	0.031	0.146
	KNC		Cumulative Years—Kinship Care	−0.049	−0.066	−0.032	0.000
	FSC		Cumulative Years—Foster Homes	0.004	−0.007	0.016	0.436
	TFC		Cumulative Years—Treatment FH	0.035	0.023	0.047	0.000
	CGC		Cumulative Years—Congregate Care	−0.045	−0.060	−0.031	0.000
	INC		Cumulative Years—Institution Care	−0.041	−0.143	0.060	0.425
Time Lag	Tau (*τ*)		Years Since the Day of Placement	0.052	0.034	0.069	0.000
Interaction of Time Lag and Placement Instability	Lag × CPC	*τ*:1-time *	Adaptation After 1 Placement	0.000	0.000	0.000	
	Lag × CPC	*τ*:2-time	Adaptation After 2 Placements	−0.020	−0.045	0.005	0.120
	Lag × CPC	*τ*:3-time	Adaptation After 3 Placements	−0.035	−0.067	−0.003	0.034
	Lag × CPC	*τ*:4+ time	Adaptation After 4 or more Placements	−0.048	−0.082	−0.014	0.006

The above table and Supplement “LMM Results” shows the result of the time-lagged longitudinal analysis produced by fitting a linear mixed model of sleep status (at least 30 days after the placement date) with the following main predictor variables: (1) placement instability quantified as cumulative placement count (CPC) so far at the time of each assessment, and (2) placement type quantified as cumulative years spent in each placement setting prior to each assessment, while controlling for (a) individual demographics and baseline traits summarized by factor analysis, (b) the autoregressive effect of sleep status on/near the day of placement, (c) years elapsed since the placement (i.e., time lag), and (d) the interaction between time lag and placement instability. We say the analysis was “time-lagged” because the primary data points were CANS assessments taken (at least 30 days) after a placement, with one exception that the sleep status at the time of the placement (Sleep_0) was appended as an autoregressive predictor to control for mutual effects between sleep status and the placement, so the effect found for other predictors is casual. Categorical variables are coded as nominal variables (under the column “category”), and a reference level is highlighted by “*”; for Race and Ethnicity, we choose “N/A” (Refused/Unknown) as the reference level to preserve analytical samples. The beta coefficient (column “beta”) shows the expected change in ratings of the “Sleep” item given one unit increase in the corresponding numerical variable, or given a particular level of a categorical variable vs. the reference level. For the current study, the most important coefficients are those with variable “CFC” (cumulative placement count), which shows the expected increase in “Sleep” rating among individuals experiencing placement instability from 2, 3, or 4+ placements vs. a single placement (i.e., stable placement) is 0.025, 0.045, and 0.067, respectively.

## Data Availability

The data used in this study are not publicly available due to restrictions imposed by the child welfare authority to protect participant confidentiality but are available from the corresponding author upon reasonable request and with permission from the data-providing agency.

## Supplementary Materials

The following supporting information can be downloaded at: https://www.mdpi.com/article/10.3390/children13050631/s1, Table S1: Data Example; Table S2: Dictionary of CANS; Table S3: LMM Results.

## Appendix A

### Appendix A.1. Data Selection and Quality Control

We received data containing 138,345 CANS assessments administered to 29,915 individuals from 1 February 2010, to 31 December 2020, and another data documenting 166,780 placement period involving 51,405 individuals from 1 May, 11,997, to 31 December 2020.

Merging the two data by placement ID (PID) reduced the data to M = 115,060 CANS assessments scattered across Q = 63,352 placement periods over N = 26,128 individuals. The reduction is due to individuals entering into the system before the CANS becoming a mandated routine.

We concatenated multiple placements with short spans but the same type, the same provider, and connected dates into a single, longer period. Doing so reduced the number of placements to Q = 55,331 and increased the mean length of placement (LOP) from 403 to 506 (days).

Even after the concatenation, there are still placements as short as a single day to less than a month. We intended to ignore short placements unlikely due to active care planning but response to unplanned emergencies (i.e., hospitalization), therefore we removed placements shorter than 30 days. As a result, the data reduced to M = 10,8420 CANS assessments covering Q = 50,935 placement periods of N = 25,392 individuals. Here, CANS assessments were reduced due to the removal of placement periods they reside, and some individuals were removed because all placements and assessments and of the said individuals were removed.

The study relies on the accurate capture of individual status by CANS (i.e., sleep status) at the beginning of each placement, especially the first placement (i.e., entry point of the out-of-home care), however, the timing of a placement was not always aligned with a CANS assessment (29% matched by exact date, 45% matched within ±30 d). Per-individual, we identify the baseline CANS as the one closest to the first placement by date, and removed 3 CANS assessment recorded prior to the baseline, which we suspect to be rare data entry errors; we then removed entire individuals whose baseline CANS fell outside of ±30 days around the first placement, which significantly shrunk the data size to M = 90,869 CANS assessments aligned with Q = 43,115 placement periods of N = 20,902 individuals.

Lastly, we exclude 3 individuals who did not report a gender, and those with an age fell out of the range of 5 to 18 years. We were left with **M = 90,827** CANS assessments aligned with **Q = 43,094** placement periods of **N = 20,888** individuals.

To be noted, from this point on, no individuals will be removed, but the downstream analysis can further reduced the number of placement and assessment: (1) per-individual, except for the first placement, a subsequent placement period is removed if none of the CANS is close enough (i.e., ±30 days) to the beginning of the said period; (2) per-placement period, the initial assessment is taken out and paired with rest of the assessment to model the auto-regressive effect of sleep, which in turn supports casual inference.

### Appendix A.2. Handling of Missing Values

Due to the substantial numbers of missingness in race and ethnicity types, we treated missing values as the reference level in their categorical variable coding, so the sample size can be maximized. The effect of being a particular race, for example, the “White”, can be interpreted as the odds ratio of observing sleep disturbance among “White” individuals versus those who did not report a race.

For gender, which has as few as 20 individuals with missing values, we removed those individuals from the data.

For CANS, there are two types of domains: (1) the “core” domains contain items mandatorily assessed and have near 0 missing values; the special “modules” however, were assessed only when a “trigger” item in one of the core domains was rated above 0. We therefore fill up missing module items with 0 if their corresponding trigger items were rated 0. Items remined with non-zero missing were “Victims of Sex Trafficking” (15.3%) and all 23 items (all 10.3%) under domain “Identified Permanent Resource Needs and Strengths”. We excluded them from the PCA. (The exact missing rate before and after 0-fill module items is available in Supplementary Materials: CANS Dictionary.

## Appendix B

### Reproducibility Justification

In this study, we chose to group all races, except for Black and White, into a category labeled ‘Other’ due to the limited number of youths from these racial backgrounds. The sample size for each of these racial groups was insufficient for meaningful separate analysis, and such aggregation was necessary to protect the privacy and confidentiality of our participants. By doing so, we aimed to minimize the risk of identifying individual participants based on their demographic characteristics and ensure the robustness of our findings by focusing on the larger and more representative categories of Black and White. This approach allowed us to maintain the integrity of the data while adhering to ethical research practices.
